# Effects of Feeder Cells on Dopaminergic Differentiation of Human Embryonic Stem Cells

**DOI:** 10.3389/fncel.2016.00291

**Published:** 2016-12-20

**Authors:** Zhenqiang Zhao, Yanlin Ma, Zhibin Chen, Qian Liu, Qi Li, Deyan Kong, Kunxiong Yuan, Lan Hu, Tan Wang, Xiaowu Chen, Yanan Peng, Weimin Jiang, Yanhong Yu, Xinfeng Liu

**Affiliations:** ^1^Department of Neurology, Jinling Hospital, Southern Medical UniversityNanjing, China; ^2^Department of Neurology, First Affiliated Hospital, Hainan Medical UniversityHaikou, China; ^3^Department of Obstetrics and Gynecology, Nanfang Hospital, Southern Medical UniversityGuangzhou, China; ^4^Hainan Provincial Key Laboratory for Human Reproductive Medicine and Genetic Research, Hainan Reproductive Medical Center, First Affiliated Hospital, Hainan Medical UniversityHaikou, China; ^5^Department of Neurology, Affiliated Ruikang Hospital, Guangxi Traditional Chinese Medical UniversityNanning, China; ^6^Department of Neurology, Central HospitalShenzhen, China; ^7^Department of Laboratory Medicines, First Affiliated Hospital, Hainan Medical UniversityHaikou, China

**Keywords:** human embryonic stem cells, neural differentiation, dopaminergic neuron, mouse embryonic fibroblasts, human foreskin fibroblasts, electrophysiological properties

## Abstract

Mouse embryonic fibroblasts (MEFs) and human foreskin fibroblasts (HFFs) are used for the culture of human embryonic stem cells (hESCs). MEFs and HFFs differed in their capacity to support the proliferation and pluripotency of hESCs and could affect cardiac differentiation potential of hESCs. The aim of this study was to evaluate the effect of MEFs and HFFs feeders on dopaminergic differentiation of hESCs lines. To minimize the impact of culture condition variation, two hESCs lines were cultured on mixed feeder cells (MFCs, MEFs: HFFs = 1:1) and HFFs feeder, respectively, and then were differentiated into dopaminergic (DA) neurons under the identical protocol. Dopaminergic differentiation was evaluated by immunocytochemistry, quantitative fluorescent real-time PCR, transmission and scanning electron microscopy, and patch clamp. Our results demonstrated that these hESCs-derived neurons were genuine and functional DA neurons. However, compared to hESCs line on MFCs feeder, hESCs line on HFFs feeder had a higher proportion of tyrosine hydroxylase (TH) positive cells and expressed higher levels of FOXA2, PITX3, NURR1, and TH genes. In addition, the values of threshold intensity and threshold membrane potential of DA neurons from hESCs line on HFFs feeder were lower than those of DA neurons from hESCs line on the MFCs feeder. In conclusion, HFFs feeder not only facilitated the differentiation of hESCs cells into dopaminergic neurons, but also induced hESCs-derived DA neurons to express higher electrophysiological excitability. Therefore, feeder cells could affect not only dopaminergic differentiation potential of different hESCs lines, but also electrophysiological properties of hESCs-derived DA neurons.

## Introduction

Parkinson’s disease (PD) is a chronically and progressively neurodegenerative disorder, and it is characterized by loss of the dopaminergic (DA) neurons in the substantial nigra pars compacta (SNpc) (Damier et al., 1999). The current PD treatments can only partially alleviate symptom but cannot completely halt the progression of neurodegeneration (Huot et al., 2013). Human embryonic stem cells (hESCs) provide a potential resource for cells transplantation to replace the extensive loss of dopaminergic neurons. Recently, many studies have demonstrated that hESCs-derived DA neurons survived, innervated, integrated, and promoted functional recovery in animal models of PD (Kriks et al., 2011; Kirkeby et al., 2012; Grealish et al., 2014; Steinbeck et al., 2015).

Dopaminergic neurons in the midbrain are divided into three distinct clusters termed the A8, A9, and A10 groups (Dahlstroem and Fuxe, 1964). Different midbrain DA neurons project to distinct regions and modulate specific functions. DA neurons in SNpc (A9 group) projected to the dorsal striatum and control voluntary movement, and the loss of these neurons is the pathological hallmark of PD (Lees et al., 2009). DA neurons in the ventral tegmental area (VTA, or A10 group) and the retrorubral field (RrF, or A8 group) projected to the ventromedial striatum, limbic areas, and prefrontal cortex, influencing will, memory and reward (Tzschentke and Schmidt, 2000). Electrophysiological characteristics of A9 DA neurons are rather typical, including a spontaneous pacemaker-like depolarizing potential, long-duration action potentials (APs), and the hyperpolarization activated inwardly rectifying cation current (Grace and Bunney, 1984a,b; Grace and Onn, 1989; Hainsworth et al., 1991; Ungless and Grace, 2012). DA neurons exhibited altered firing patterns, decreased cell capacitance and dopamine releases, and disrupted pacemaker firing regularity in the MitoPark mouse model of PD, suggesting that electrophysiological functional adjustment occurred in single neurons before the overt motor impairment (Good et al., 2011; Branch et al., 2016). Therefore, it is important to understand the electrophysiological properties of hESCs-derived DA neurons before they are used for transplantation. To date, the electrophysiological criteria of hESCs-derived DA neurons are still not established.

Feeder cells play a crucial role in the establishment of hESCs lines and the maintenance of hESCs growth and pluripotent status. The first hESCs line was originally derived and cultured on mouse embryonic fibroblasts (MEFs) feeder cells in Thomson et al. (1998). MEFs cells secrete various growth factors and cytokines to support the pluripotency and proliferation of hESCs in long-term period, such as TGFβ1and activin A (Eiselleova et al., 2008). However, MEFs could transfer animal-derived infectious pathogens and increase the risk of an immune response after hESCs transplantation, such as viral particles and sialic acid Neu5GC (Martin et al., 2005; Cobo et al., 2008). For replacing animal cells, various alternative human feeder layers such as fetal foreskin, fetal muscle, and skin (Richards et al., 2002; Amit et al., 2003), have been validated for culture of undifferentiated hESCs. Human foreskin fibroblasts (HFFs) feeder cells have been used to establish the new hESCs lines (Aguilar-Gallardo et al., 2010). Moreover, MEFs and HFFs differed in their capacity to support the pluripotency and non-differentiation of hESCs. HFFs supported mouse ESCs self-renewal superiorly to MEFs because HFFs secreted substantial quantities of bFGF, while no bFGF was secreted by the MEFs. (Eiselleova et al., 2008; Yang et al., 2016). In addition, differences in feeder cells also had a considerable influence on the cardiac differentiation potential of the human pluripotent stem cells (hPSCs) (Ojala et al., 2012; Pekkanen-Mattila et al., 2012).

In recent years, most of hESCs lines used for the generation of dopaminergic (DA) neurons are derived on MEFs, such as H1 (Kriks et al., 2011), H9 (Kirkeby et al., 2012), H14 (Momcilovic et al., 2014). Limited data are available regarding functional differentiation of DA neurons derived from hESCs lines on HFFs. Furthermore, whether the differences in feeder cells have significant impacts on the functional differentiation of dopaminergic neurons of hESCs lines remain unclear. Therefore, we try to investigate functional differentiation of DA neurons derived from hESCs cell lines derived on different feeder cells for clinical applications.

In order to minimize the impact of culture condition variation on dopaminergic differentiation of individual hESCs line, we adapted our hESCs line, HN4 to mixed feeder cells (MFCs, a mixture of MEFs and HFFs feeder, MEFs: HFFs = 1:1), and P96 to HFFs feeder cells. The whole process of differentiation was performed by identical differentiation protocol based on the application of SB431542 (SB) and Dorsomorphin (DM). In this study, we aimed to systematically investigate the effect of MEFs and HFFs feeder on dopaminergic differentiation of hESCs lines including neural induction process, differentiation potential, morphology and electrophysiological properties of hESCs-derived DA neurons. Our findings suggested that feeder cells affected not only dopaminergic differentiation potential of hESCs line, but also electrophysiological functions of the hESCs-derived DA neurons.

## Materials and Methods

All samples were collected following protocols approved by the Ethics Committee of Hainan Provincial Reproductive Medical Center of Hainan Medical University. All human volunteers have provided their written informed consent and the Ethics Committees have approved this consent procedure.

### Generation and Culture of hESCs Lines

The hESCs lines HN4 and P96 were established in Hainan Provincial Reproductive Medical Center of Hainan Medical University. The HN4 cell line was cultured originally on mixed MEFs and HFFs feeder (MFCs, MEFs: HFFs = 1:1) as previously described (Li et al., 2010; Liu et al., 2013). P96 cell line was cultured originally on HFFs with mTeSR1 medium (STEMCELL Technologies, CA). Inner cell mass (ICM) and HFFs were isolated and cultured as described previously (Li et al., 2010). The characterization of totipotency and multi-lineage differentiation potential of P96 cell line were performed by Alkaline Phosphatase staining, Karyotype analysis, immunocytochemistry, quantitative real time RT-PCR, embryoid body (EB) formation *in vitro* and teratomas formation *in vivo* in our institution as described previously (Li et al., 2010). To adapt to the new culture system, the two cell lines were cultured and maintained on Matrigel-coated 6-well culture plates (BD Biosciences, USA) with mTeSR1 media before differentiation. Cell culture medium was changed every day and cells were passaged every 5 days. The hESCs were used for further experiments after three or more passages in cell cultures.

### Dopaminergic Differentiation of hESCs

Human embryonic stem cells were seeded on Matrigel coated 6-well culture plates at a density of 4 × 10^4^ cells/cm^2^ and cultured for 48 h to reach 80 ∼ 90% confluence. For neural differentiation, hESCs were cultured in Neural Maintenance Medium (NMM) supplemented with 5 μM of TGF-β inhibitor SB431542 (SB, Selleckchem, USA) and 1 μM of bone morphogenetic protein (BMP) inhibitor Dorsomorphin (DM, Selleckchem, USA) (Shi et al., 2012). After 8 days, the cells were cultured in NMM without SB and DM for 8 days. Neural progenitor cells were manually passaged and replanted onto poly-D-lysine/laminin-coated plates in NMM supplemented with 0.2 mM vitamin C (Sigma–Aldrich, USA), 100 ng/ml sonic hedgehog (SHH, R&D Systems, USA) and 100 ng/ml fibroblast growth factor-8b (FGF8b, Peprotech, USA) for 10 days. Neurons were matured for an additional 2 weeks in NMM supplemented with 10 ng/ml brain-derived neurotrophic factor (BDNF, R&D Systems, USA), 10 ng/ml glial cell line-derived neurotrophic factor (GDNF, R&D Systems, USA), 10 ng/ml insulin-like growth factor 1 (IGF1, Peprotech, USA), 500 μM cyclic adenosine monophosphate (cAMP, Sigma, USA). Half of the cell culture medium was replenished every other day.

### Immunocytochemistry and Cell Counting

Differentiated cells were fixed for 30 min with 4% paraformaldehyde, and blocked with 5% normal goat serum and 1% BSA in 0.2% Triton X-100 for 45 min. Primary antibodies were diluted in 5% normal goat serum and incubated with the samples overnight at 4°C. The appropriate fluorescently labeled secondary antibodies were applied for 2 h at room temperature. The nuclei were counter stained with 4, 6-diamidinodiamidino-2-phenylindole (DAPI, 10 mg/ml, Life Technologies). Negative control (omit primary antibody) was included in all immunofluorescent staining. Immuno labeled cells were viewed and counted using Zeiss LSM 710 NLO laser scanning confocal microscope (Jena, Germany). The percentage of MAP-2/TH/DAPI positive cells was calculated within 10 randomly selected visual fields. The following primary antibodies were used: 1:500 rabbit anti-TH (Millipore, AB5935), 1:500 mouse anti-MAP2 (Abcam, ab11267) 1:200 goat anti-GIRK2 (Abcam, ab65096). The secondary antibodies were as follows: Alexa Fluor 488 goat anti-mouse (1:400, ab150113, Abcam), Alexa Fluor 488 donkey anti-goat (1:400, ab150129, Abcam) and Alexa fluor 594 goat anti-rabbit (1:400, ab150080, Abcam).

### Quantitative Real Time RT-PCR (qRT-PCR)

Total RNA was extracted from cultured cells using RNeasy MicroKit (Qiagen, Germany) and treated with DNase according to manufacturer’s instructions. For each reaction, 2 μg of total RNA was reversely transcribed using oligo-dT primers and Superscript II reverse transcriptase (Thermo Fisher Scientific, USA). Real-time PCR analysis was performed by CFX96 Real-Time PCR system (Bio-Rad IQ5, Hercules, CA, USA) and SYBR Green PCR Master Mix (Thermo Fisher Scientific, USA). All primer sequences were listed in **Table 1**. β-actin was used as a reference gene. Relative expression ratios were calculated using Pfaffl’s calculations based on the ^ΔΔ^Ct method (Pfaffl, 2001). The changes of all genes of interest in the HN4-derived cell sample were calculated relative to P96-derived cell sample.

**Table 1:** Primers used for quantitative fluorescent real-time PCR (qRT-PCR) analysis during neural differentiation of human embryonic stem cells (hESCs).

Genes	Primer sequences
FOXA2 Forward Primer	5′-CTGAGGCCCACCTGAAGCC-3′
FOXA2 Reverse Primer	5′-GTAGCCGGGGTAGTGCATCA-3′
LMX1A Forward Primer	5′-GCAAAGGGGACTATGAGAAGGA-3′
LMX1A Reverse Primer	5′-CGTTTGGGGCGCTTATGGT-3′
TH Forward Primer	5′-GGGCTGTGTAAGCAGAACG-3′
TH Reverse Primer	5′-AAGGCCCGAATCTCAGGCT-3′
GIRK2 Forward Primer	5′-CACATCAGCCGAGATCGGAC-3′
GIRK2 Reverse Primer	5′-GGTAGCGATAGGTCTCCCTCA-3′
PITX3 Forward Primer	5′-CCTACGAGGAGGTGTACCCC-3′
PITX3 Reverse Primer	5′-AGGCGAATGGAAAGGTCTTGG-3′
EN1 Forward Primer	5′-CGCAGCAGCCTCTCGTATG-3′
EN1 Reverse Primer	5′-CCTGGAACTCCGCCTTGAG-3′
NURR1 Forward Primer	5′-ACCACTCTTCGGGAGAATACA-3′
NURR1 Reverse Primer	5′-GGCATTTGGTACAAGCAAGGT-3′
NCAM Forward Primer	5′-GGCATTTACAAGTGTGTGGTTAC-3′
NCAM Reverse Primer	5′-TTGGCGCATTCTTGAACATGA-3′
β-Actin Forward Primer	5′-TTAGTTGCGTTACACCCTTTCTTGACA-3′
β-Actin Reverse Primer	5′-CTGTCACCTTCACCGTTCCAGTTTT-3′


### Electron Microscopy

For scanning electron microscopy (SEM) analysis, hESCs were cultured on 5 mm in diameter glass coverslip. At the end of dopaminergic differentiation, specimens were fixed in cold 2.5% glutaraldehyde for 120 min, post fixed in 2% osmium tetroxide (EMS) for 60 min, and dehydrated using gradually increased concentrations of ethanol. Specimens were then critical point dried from liquid CO_2_ and sputter coated with 5 nm gold/palladium. Cells were examined by SEM (Hitachi S3000N, Japan) at 20 kV accelerating voltage.

For transmission electron microscope (TEM), the cell samples were fixed in 2.0% glutaraldehyde for 2 h. Subsequently, the samples were washed for two times and post fixed with 1% osmium tetroxide for 1.5 h, and dehydrated using gradually increased concentrations of ethanol. Finally, the specimens were embedded in epoxy resin, sectioned at 70 nm thicknesses with an ultra-microtome, stained with uranyl acetate and lead citrate for 8 min, and examined by transmission electron microscopy (Hitachi H7500, Japan).

### Electrophysiology Recording

In order to investigate the electrophysiological properties of hESCs-derived DA neurons, electrophysiology recording was carried out using patch clamp. For patch clamp, neuronal-like cells with a large and angular morphology were selected for recording at room temperature (22–25°C). Pipettes (initial resistance 2–5 MΩ) were pulled from borosilicate glass (Clarke, UK) using a P87 puller (Sutter Instruments, USA). An Axopatch 200B patch clamp amplifier in conjunction with a Digidata 1400 interface (Molecular Devices, USA) was used for recording. For recording spontaneous postsynaptic currents (PSCs), ligand- and voltage-gated ion channels currents, neuronal-like cells were held at -70 mV in voltage-clamp mode. For evoking APs, resting potentials of neuronal-like cells were maintained at about -60 mV in current-clamp mode and stimulated with depolarizing current injections. Graded current injections used durations of 300 ms (in increments of 5 pA). For the recording of voltage-gated total ionic currents, AP_S_, ligand-gated currents and spontaneous activity, the bath solution contained (in mM): 120 NaCl, 1.2 KH_2_PO4, 1.9 KCl, 26 NaHCO_3_, 2.2 CaCl_2_, 1.4 MgSO_4_,10 D-glucose, 7.5 HEPES, pH 7.3 adjusted with NaOH. The pipette solution contained (in mM): 140 potassium methanesulfonate, 10 HEPES, 5 NaCl, 1 CaCl_2_, 0.2 EGTA, 3 ATP-Na_2_, 0.4 GTP-Na_2_, pH 7.3 adjusted with KOH. For further ensure the sodium channels, the calcium currents were blocked by the addition of 0.2 mM CdCl_2_, and then the sodium currents were blocked by the additions of 10 μM tetrodotoxin (TTX) to the bath solution. To investigate the potassium channels, potassium methanesulfonate was replaced by 140 mM CsCl in pipette solution, and then 10 μM tetraethylammonium (TEA) and 3 mM 4-aminopyridine (4-AP) were added into the bath solution to block the potassium currents. Drug Applications: Receptor agonists 1mM Glutamate and 1mM γ- aminobutyric acid (GABA) were applied by pressure pulses (10 psi, 100msec in duration). Data analysis were carried out using pCLAMP 10.2 (Axon Instruments Inc.) and ORIGIN 8.0 (OriginLab Corp, Northampton, MA, USA).

### Statistical Analysis

Results were presented as mean ± SEM unless otherwise stated. Comparison analysis was performed with Student’s *t-*test. The relationship between the number of spikes elicited and the intensity of the depolarizing currents was analyzed by Pearson correlation coefficient (Pearson’s *r*-value). All of the statistical tests were performed using the Statistical Package SPSS version 19.0. Significance levels were set to *P* < 0.05 for all comparisons.

## Results

### Generation and Adaptation Culture of hESCs Lines

**FIGURE 1:**
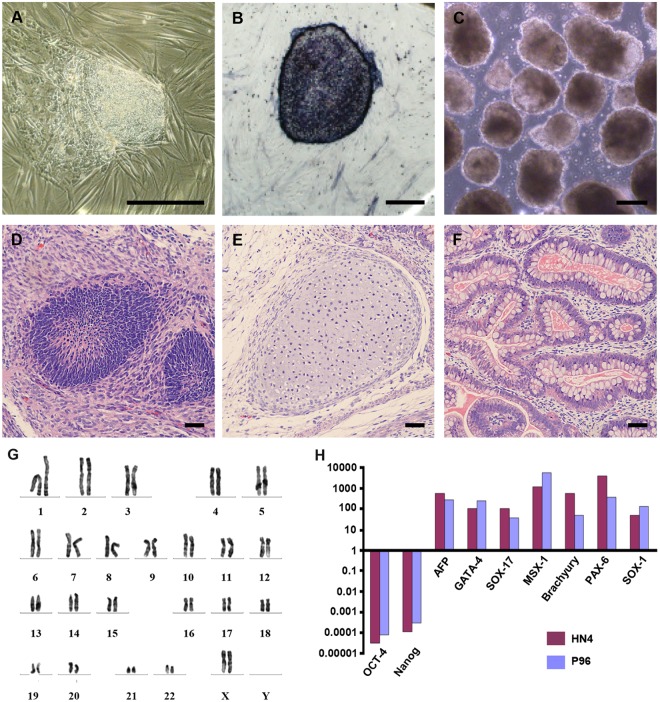
**Generation and identification of P96 human embryonic stem cell (hESCs) line.** **(A)** A primitive colony of P96 cell line on day 4 after secondary isolation. Scale bars = 200 μm. **(B)** Alkaline Phosphatase staining of P96 cell line colony. Scale bars = 200 μm. **(C)** Embryoid body formation to detect totipotent potential of P96 cell line *in vitro*. Scale bars = 500 μm. **(D–F)** H and E staining of the teratomas formed by P96 cell line for 28 passages. The hESCs could be differentiated into three different germ layers: endoderm **(D)**, mesoderm **(E)**, and ectoderm **(F)**. Scale bars = 200 μm. **(G)** A normal karyotype analysis of P96 cell line at the passage 25 (46, XX). **(H)** Real time RT-PCR to detect the makers of three different germ layers from HN4 and P96 cell line. ESC markers (OCT-4, Nanog); Endoderm markers (AFP, GATA-4, SOX-17); Mesoderm markers (MSX-1, Brachyury); Ectoderm markers (PAX-6, SOX-1).

**FIGURE 2:**
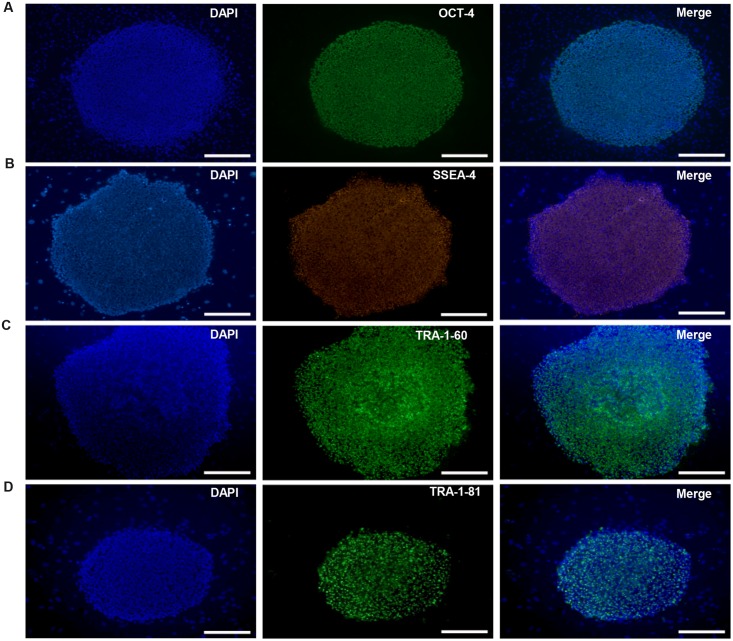
**Immunocytochemical analysis of P96 cell line at passage 38.** Immunofluorescence staining of undifferentiated P96 cells using OCT-4 **(A)**, SSEA-4 **(B)**, TRA-1-60 **(C)**, and TRA-1-81 **(D)**. Scale bars = 200 μm.

HN4 cell line was cultured originally on MFCs feeder as previously described (Li et al., 2010). The primitive P96 hESCs colony was observed in 5–7 days after the secondary isolation (**Figure 1A**). The new ES cells were perfectly homogenous and had been cultured for more than 30 passages *in vitro*. To verify the pluripotency of these new hESCs lines, markers of stemness were detected by immunostaining and Real time-PCR. These new hESCs lines expressed a very high level alkaline phosphatase activity (**Figure 1B**). Karyotype analysis was carried out to test the genetic stability of these cell lines after long-term culture. We detected the differentiation potential of these cell lines both *in vitro* and *in vivo*. The differentiation potential of P96 was assayed via EB formation *in vitro* (**Figure 1C**). *In vivo*, we applied the teratoma assay to examine the totipotency of these cell lines. The appearances of endodermal, mesodermal, and ectodermal tissues in the teratomas demonstrated the characteristic differentiation capacity of these cell lines (**Figures 1D–F**). G-banding technique was applied to examine the karyotype and the results showed that these new hESCs lines had a normal karyotype (**Figure 1G**). Meanwhile, In EB assay, the gene expression of AFP, GATA4, SOX17, MSX-1, Brachyury T, PAX-6, and SOX1 indicated that the two cell lines could differentiate into all three embryonic germ layers (**Figure 1H**). Pluripotent marker genes OCT-4, SSEA-4, TRA-1-60, and TRA-1-81 were detected in our research and all hESCs lines expressed these stem genes perfectly (**Figures 2A–D**).

### Effects of Feeder Cells on Dopaminergic Differentiation

**FIGURE 3:**
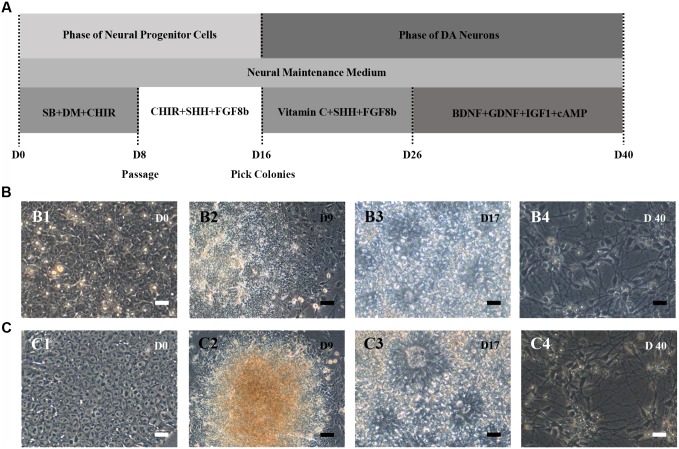
**Schematic depicting of dopaminergic (DA) neuronal differentiation from different hESCs lines.** **(A)** Flow diagram of the differentiation protocol with addition of growth factors/small molecules. **(B,C)** Morphological changes during neural differentiation. P96 **(B)** and HN4 **(C)** cell lines experienced a similar transformation process. **(B1,C1)** The hESCs cells were cultured onto Matrigel-coated dish and exhibited cobblestone-like shape. **(B2,C2)** A differentiating human embryonic stem cell colony was observed in HN4 and P96 cells lines. These cells extended short processes and gradually lose hESCs morphology on Day 9. **(B3,C3)** Neural progenitor cells exhibited neural rosette-like structures on Day 17. **(B4,C4)** At the end of differentiation, these cells exhibited typical neuronal morphology and formed elaborate neural network on Day 40. Scale bar = 50 μm.

**FIGURE 4:**
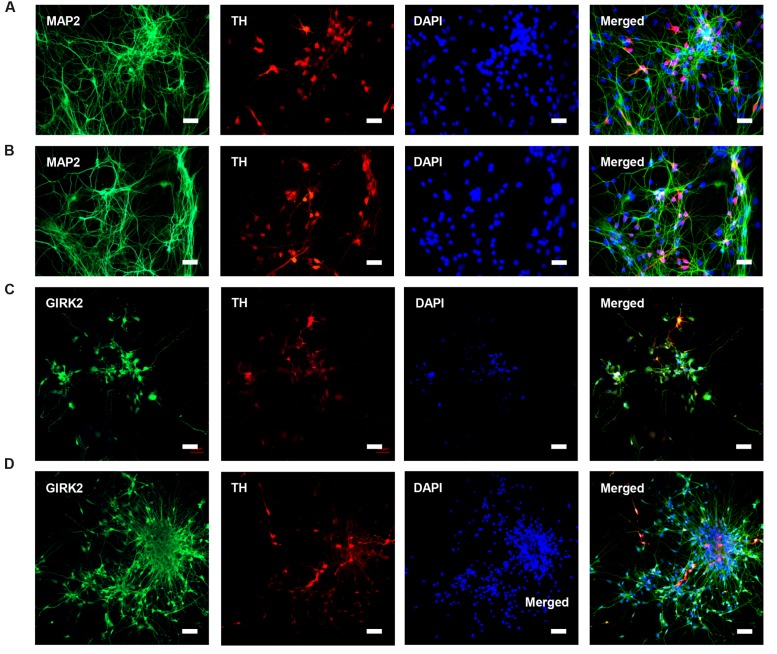
**Characteristics of DA neurons from P96 and HN4 hESCs lines.** **(A,B)** P96 **(A)** and HN4 cell line **(B)** could be differentiated into DA, which co-expressed dopaminergic neuronal marker TH+/MAP2+. These cells were gathered in clusters, and neurites from different clusters formed some connections. **(C,D)** DA neurons from P96 **(C)** and HN4 cell line **(D)** co-expressed TH+/GIRK2+, which were A9 DA neurons. Scale bar = 50 μm.

HN4 and P96 hESCs were differentiated using a simplified monolayer method. An overview of the differentiation protocol is shown in **Figure 3A**. To identify the effect of feeder cells on neural induction process, we first compared the morphology changes of HN4 and P96 hESCs lines. Undifferentiated hESCs exhibited cobblestone-like shape with small cell body and high nuclear-to-cytoplasm ratio (**Figures 3B1,C1**). Neural induction was initiated by a dual SMAD inhibition strategy using SB and DM. Both HN4 and P96 cells extended short processes and gradually lose hESCs morphology on Day 9 (**Figures 3B2,C2**), and exhibited neural rosette structures on Day 17 (**Figures 3B3,C3**). Next, expression of midbrain pattern of neural progenitor cells was induced by vitamin C, SHH, and FGF8b for 10 days. At the end stage of differentiation, Neuronal maturation was achieved through addition of BDNF, GDNF, IGF1, and cAMP. As differentiation continued, these cells gradually exhibited typical neuron morphology. On Day 40, differentiated cells showed large, angular or fusiform morphologies with lots of neurites (**Figures 3B4,C4**). These cells aggregated as small clusters, and neurites from different clusters formed elaborate neural networks. Extensive immunocytochemical analysis was performed on differentiated cells and clusters of differentiated neurons expressed neuronal markers MAP2, were found in differentiating population of HN4 and P96 cell lines. In addition, some MAP2-positive cells expressed dopaminergic marker tyrosine hydroxylase (TH), suggesting that these were dopaminergic neurons (**Figures 4A,B**). Furthermore, the A9 dopaminergic neuron marker, GIRK2 was also expressed in some TH-positive cells (**Figures 4C,D**).

**FIGURE 5:**
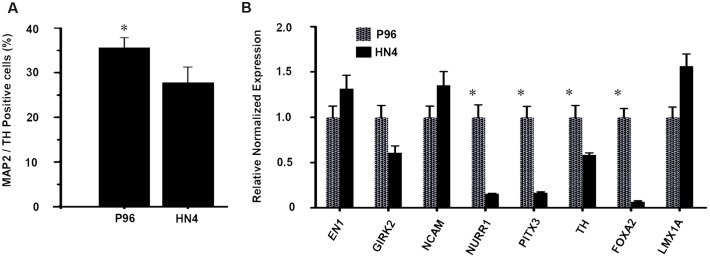
**The proportion and gene expression level of DA neurons from P96 and HN4 hESCs lines.** **(A)** At the end of differentiation, the percentage of TH+/MAP2+ cells of P96 cell sample was higher than that of HN4 cell sample (^∗^*p* < 0.05 vs. HN4). **(B)** The expression of LMX1A, NURR1, PITX3, EN1, NCAM, TH, GIRK2, and FOXA2 was measured by qRT-PCR in the differentiated samples of P96 or HN4 cell line. The expression levels of FOXA2, TH, PITX3 andNURR1 were increased in P96-derived cell population (^∗^*p* < 0.05 vs. HN4). Data presented as mean ± SEM.

Dopaminergic differentiation efficiency was quantified by cell counting. On Day 40, the proportion of MAP-2/TH-double positive cells was 35.6% ± 2.2% in P96-derived cells, while proportion was only 27.8% ± 3.5% for HN4-derived cells, suggesting a higher dopaminergic differentiation efficiency of P96 cell line than that of HN4 cell line (*P* < 0.05) (**Figure 5A**). Quantitative fluorescent real-time PCR analysis potential revealed that expression of markers of midbrain DA development, including FOXA2, LMX1A, TH, PITX3, EN1, NURR1, and NCAM, was induced during *in vitro* differentiation. Expression of the A9-type midbrain DA neuron marker Girk2 was also detected. The expression of LMX1A, GIRK2, EN1, and NCAM were similar between HN4- and P96-derived cell populations. However, P96-derived cell population exhibited significantly higher expression levels of FOXA2, TH, PITX3, and NURR1, demonstrating 14.9-fold, 1.7-fold, 5.9-fold, and 6.5-fold increase, respectively, in comparison with HN4-derived cells (**Figure 5B**). These results indicated that feeder cells had no effects on neural induction process; instead they played a significant role in dopaminergic differentiation potential of hESCs line.

### Effects of Feeder Cells on Morphology and Ultrastructure of Differentiated DA Neurons

**FIGURE 6:**
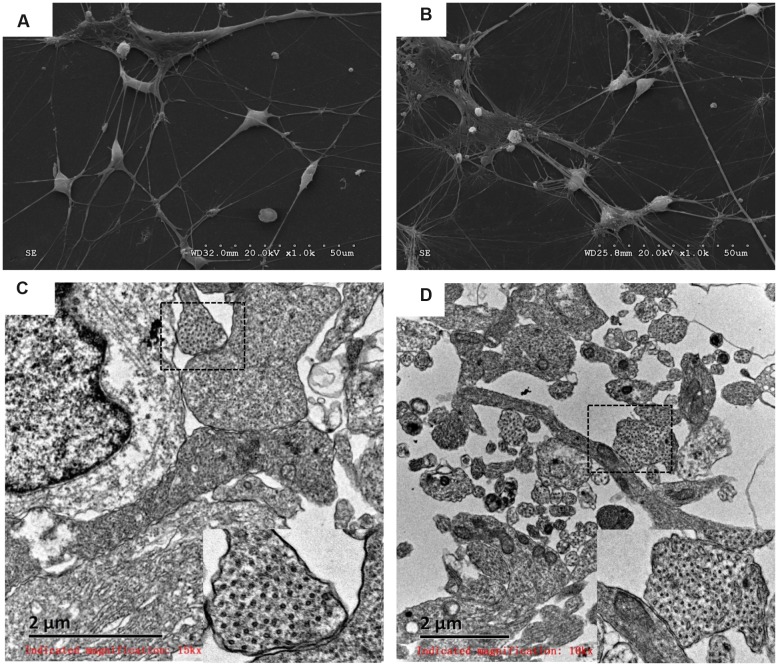
**Analysis of P96- and HN4-derived DA neurons by scanning electron micrograph and transmission electron micrograph.** **(A,B)** P96- **(A)** and HN4-derived neurons **(B)** showed the elongated, angular A9 DA neurons with laterally and ventrally projecting dendrites. **(C,D)** The ultrastructure of P96- **(C)** and HN4-derived DA neurons **(D)** showed nucleus containing euchromatin, neurofilaments, and ribosomes. The typical synaptic structure and numerous synaptic vesicles located in presynaptic zone were observed in the dashed frame.

The morphology and ultrastructure of differentiated DA neurons were investigated with SEM and TEM. Similar external morphology and internal ultrastructure were observed in HN4- and P96-derived DA neurons. Differentiated neurons aggregated into small clusters. Furthermore, many neurites emerged from these clusters, and formed elaborate networks. The shapes of neuronal cell body were spherical, ovoid, pyramidal and fusiform. The membrane surfaces of neuron were uneven. The typical morphology of DA neurons could be found in this study: elongated, angular neurons and fusiform neurons with laterally and ventrally projecting neurites, 6 ∼ 10 μm in diameter (**Figures 6A,B**); the internal ultra-structure of differentiated DA neurons and synaptic connections were analyzed with TEM. The typical structures of P96-, HN4-derived DA neurons included organelle, nucleus, ribosome, neurofilament and neurites. The typical synaptic structure and numerous synaptic vesicles located in presynaptic zone were observed in both P96- and HN4-derived DA neurons (**Figures 6C,D**). These results indicated that feeder cells had no effects on the morphology and structure of differentiated DA neurons themselves.

### Effects of Feeder Cells on Electrophysiological Properties of Differentiated DA Neurons

Electrophysiological properties of HN4-, P96-derived DA neurons were investigated *in vitro*. Voltage-and ligand-gated channels, APs and synaptic activity were investigated with whole-cell current and voltage clamp techniques.

**FIGURE 7:**
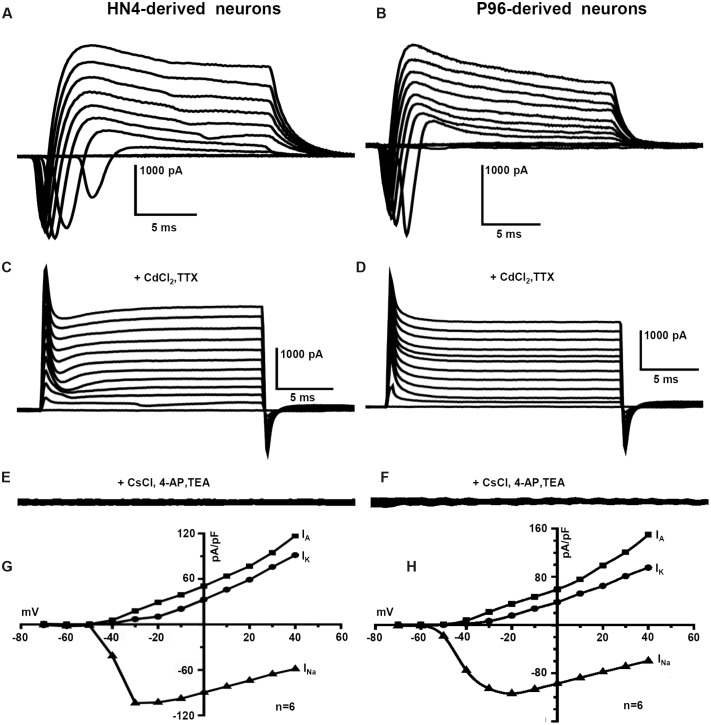
**Voltage-gated ion channels currents of P96- and HN4-derived DA neurons.** Voltage-activated sodium and potassium currents were recorded in the whole-cell voltage-clamp mode by increasing depolarizing steps of 10 mV from a holding potential of -70 to 40 mV. **(A,B)** The typical whole cell currents recorded in P96- **(A)** or HN4-derived neurons **(B)** exhibited three major components: transient inward current, transient outward current, and delayed rectifier current. **(C,D)** All inward currents were blocked by the addition of 10 μM TTX and 0.2 mM CdCl_2_ in P96- **(C)** or HN4-derived neurons **(D)**. **(E,F)** All outward currents were blocked by the addition of 10 μM TEA and 3 mM 4-AP and replacement of potassium methanesulfonate with CsCl in pipette solution in P96- **(E)** or HN4-derived neurons **(F)**. **(G,H)** The current-voltage plot of whole cell currents. The transient inward currents *I*_Na_ started to activate at about -60 mV in P96-derived neurons **(G)** or -50 mV in HN4-derived neurons **(H)**. The transient outward potassium currents *I*_A_ and delayed rectifier currents *I*_K_ started to activate at about -50 mV or -40 mV, respectively. The average peak current amplitudes were normalized for cell capacitances.

The capacitance of cells membrane (Cm) of HN4- and P96-derived neurons was 14.42 ± 4.1 pF and 14.40 ± 3.5 pF, respectively (*n* = 6, *P* > 0.05), (**Table 2**). The similar Voltage-gated currents were recorded in the HN4- and P96-derived neurons. A fast activating inward current, a fast inactivating outward current and a delayed rectifier outward current were evoked by depolarizing voltage steps of 10 mV from a holding potential of -70 to 40 mV (**Figures 7A,B**). All inward currents were abolished by supplement of TTX and CdCl_2_ to bath solution (**Figures 7C,D**), whereas application of 4-AP, TEA, and CsCl blocked almost all outward currents (**Figures 7E,F**). These results demonstrated that the neurons expressed voltage-gated sodium channels (*I*_Na_ currents), transient outward potassium channels (*I*_A_ currents) and delayed rectifier potassium channels (*I*_K_ currents). The current-voltage (I–V) relationships were well comparable between the two hESCs-derived DA neurons. Currents amplitudes were normalized for cell size based on the capacitance of the cell membrane (pA/pF, termed current density). In HN4- and P96-derived neurons, *I*_Na_ currents appeared at around -50 mV or -60 mV, and reached the peak at around -30 mV or -20 mV, respectively (**Figures 7G,H**). The average peak current density of HN4- and P96-derived neurons were -103.88 ± 26.88 pA/pF (*n* = 6) and -113.97 ± 22.54 pA/pF (*n* = 6), respectively. No significant difference was detected in peak current density of sodium currents (*P* > 0.05). However, the initial activated potential of sodium channels in P96-derived neurons appeared lower level than that in HN4-derived neurons. In both the hESCs-derived neurons, *I*_A_ was evoked at around -50 mV and *I*_K_ was elicited at around -40 mV (**Figures 7G,H**). The average current density of *I*_A_ at 40 mv was 149.84 ± 38.62 pA/pF (*n* = 6) for HN4-derived neurons and 104.08 ± 22.27 pA/pF (*n* = 6) for P96-derived neurons, respectively. The average current density of *I*_K_ at 40 mv was 95.16 ± 16.86 pA/pF (*n* = 6) for HN4-derived neurons and 82.37 ± 18.68 pA/pF (*n* = 6) for P96-derived neurons, respectively. No significant difference was detected regarding to potassium channel (*P* > 0.05). The results showed that the voltage-gated sodium channels of P96-derived neurons were activated more easily. Feeder cells affected the activation of voltage-gated sodium channels of differentiated DA neurons.

**Table 2:** Comparison of electrophysiological parameters of HN14- and P096-derived neurons.

Parameters	HN4 (*n* = 6)	P96 (*n* = 6)
Cm	14.42 ± 4.1 pF	14.40 ± 3.5 pF
RMP (mV)	-63.38 ± 0.01^∗^	-58.7 ± 0.04
Peak_AP_ (mV)	63.46 ± 0.51^∗^	65.24 ± 0.24
AP duration (ms)	5.8 ± 0.24^∗^	4.45 ± 0.08
Rise time	2.06 ± 0.72	1.86 ± 0.06
Decay time	3.71 ± 0.22^∗^	2.58 ± 0.40
Spike Threshold (mV)	-36.15 ± 0.34^∗^	-39.58 ± 0.17
GABA currents Amplitude (pA)	-393.28 ± 102.56	-694.26 ± 153.48
Glutamate currents Amplitude (pA)	-140 ± 36.38^∗^	-522.47 ± 126.72


*Data are means ± SEM; *n*, number of cells used for analysis. ^∗^Statistically significant (*P* < 0.05), unpaired Student’s *t*-test. Note: Cm, capacitance of membrane; RMP, resting membrane potential, measured in I = 0 mode; Peak_AP_, peak amplitude of AP, estimated by deducing the threshold value from the maximal depolarization voltage; AP duration, duration of AP, estimated as time between the start of the rising phase of the AP (threshold) and the equipotential point of the falling phase(Sorensen et al., 2005); Rise time, calculated from the threshold to the peak amplitude in the rising stage; Decay time, calculated from the peak amplitude to the equipotential point of threshold; Spike Threshold, the point where the fastest rising phase of the AP started.*

**FIGURE 8:**
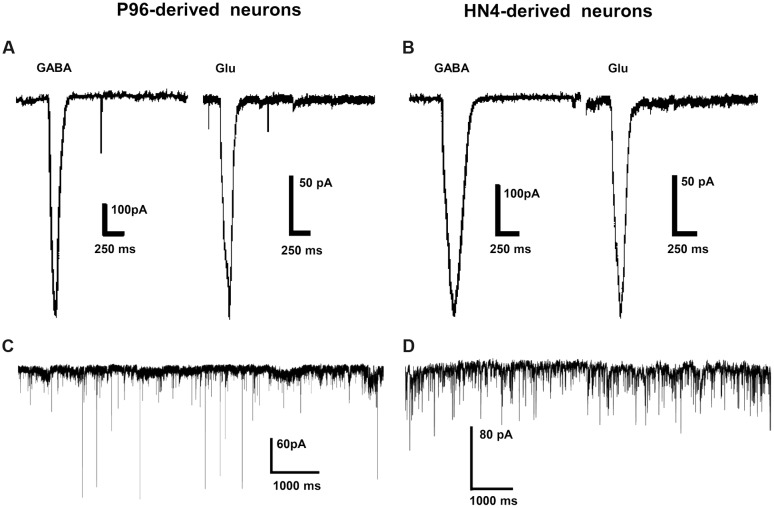
**Ligand-gated currents and spontaneous postsynaptic currents of P96- and HN4-derived DA neurons.** **(A,B)** GABA currents and Glutamate currents were recorded in P96- **(A)** or HN4-derived DA neurons **(B)** at a holding potential of -70 mV in voltage-clamp mode. **(C,D)** DA neurons from P96 **(C)** and HN4 cell line **(D)** received functional synaptic inputs, as illustrated by spontaneous postsynaptic currents (PSCs) recorded at a holding potential of -70 mV in voltage-clamp mode.

The ligand-gated currents in both HN4- and P96-derived DA neurons were recorded. The GABA ligand (1 mM) evoked a transient, slowly desensitizing inward current response at a holding potential of -70 mV. The average current amplitude was -393.28 ± 102.56 pA (*n* = 6) for HN4-derived neurons, and -694.26 ± 153.48 pA for P96-derived neurons (*n* = 6), (**Figures 8A,B**). The application of glutamate (1 mM) evoked slow inward currents in both the hESCs-derived neurons (**Figures 8A,B**). The average current amplitude was -140 ± 36.38 pA (*n* = 6) for HN4-derived neurons and -522.47 ± 126.72 pA (*n* = 6) for P96-derived neurons (**Table 2**). Our data suggested that both of hESCs-derived neurons developed functional GABA and Glutamate receptors *in vitro*. The amplitude of Glutamate-evoked currents of P96-derived DA neurons was higher than that of HN4- derived DA neurons (*P* < 0.05). The results suggested the more Glutamate receptors of P96-derived DA neurons were activated under the same concentration of Glutamate. Feeder cells affected the expression of Glutamate receptors of differentiated DA neurons.

Spontaneous synaptic activity indicated the formation of synaptic connections. We also observed the presence of synaptic vesicles at sites of contact between the two neurites by TEM. To investigate synaptic maturation in both hESCs-derived neurons, the spontaneous PSCs could be recorded in voltage clamp mode. Both HN4- and P96-derived neurons displayed functional synaptic inputs, and fired traces of spontaneous PSCs under voltage-clamp mode at a holding potential at -70 mV (**Figures 8C,D**).

**FIGURE 9:**
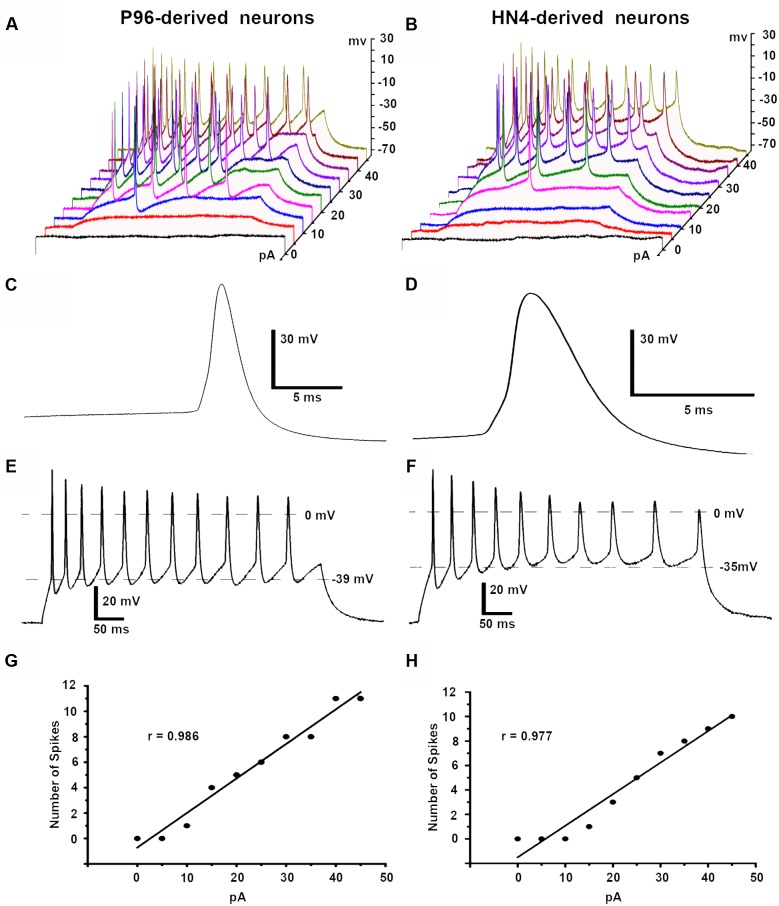
**Action potentials (APs) of P96- and HN4-derived DA neurons.** APs were elicited by a depolarizing current step protocol from 0 to 45 pA in 5 pA increments. **(A)** The first single AP was evoked by 10 pA current injections while repetitive APs were observed in response to 15 pA current injections in P96-derived neurons. **(B)** The first single AP was evoked by 15 pA current injections while repetitive APs were observed in response to 20 pA current injections in HN4-derived neurons. **(C,D)** Analysis of the first single AP of P96- **(C)** and HN4-derived neurons **(D)** showed typical AP waveform of DA neurons. The duration of the APs of P96- and HN4-derived DA neurons were >2 ms, which met the electrophysiological criteria of DA neurons. **(E,F)** The specific repetitive burst firing patterns in response to 45 pA current injection were observed in P96- **(E)** or HN4-derived neurons **(F)**. The AP train revealed that each successive spike was a progressive increase in AP duration and spike threshold, a decline in AP amplitude. **(G,H)** A linear relationship between the number of spikes elicited and the intensity of the depolarizing stimulus was found in P96- **(G)** or HN4-derived DA neurons **(H)**.

Both HN4- and P96-derived DA neurons were able to trigger serials of the APs by a depolarizing current step of 5 pA ranging from 0 to 45 pA in current-clamp mode. The first single AP of P96-derived DA neurons were evoked by 10 pA injection current, while it was 15 pA for HN4- derived DA neurons. Repetitive AP traces of P96-derived DA neurons were evoked by 15 pA injection current, while it was 20 pA for HN4- derived DA neurons (**Figures 9A,B**). The shape of APs of the two hESCs-derived neurons demonstrated a typical waveform of DA neurons, and the duration of AP was both >2 ms (**Figures 9C,D**). Both P96- and HN4-derived DA neurons have been detected to be triggered in a bursting pattern with consecutive spikes in burst displaying progressively decreasing amplitude and increasing duration (**Figures 9E,F**). A linear relationship was found between the number of spikes elicited and the intensity of the depolarizing currents (**Figures 9G,H**). To further compare electrophysiological features of AP, the AP parameters were measured including resting membrane potential (RMP), peak amplitude of AP (Peak_AP_), AP duration, rise time, decay time, and spike threshold potential. These results indicated that the differentiated cells exhibited the characteristics of neurons. Except for the rise time (*P* = 0.055), significant differences in these electrophysiological parameters of first spike of AP traces were displayed between the two hESCs-derived neurons (*n* = 6, *P* < 0.05) (**Table 2**). The results suggested the threshold intensity and threshold potential of P96-derived DA neurons were lower than that of HN4-derived DA neurons. Feeder cells affected the excitability of differentiated DA neurons.

## Discussion

In general, hESCs could be cultured under variable conditions. Maintenance of feeder layers is very labor intensive with lot-to-lot variation. This fluctuation can ultimately affect the stem cells and their downstream differentiation into specific cell types for clinical therapy (Skottman and Hovatta, 2006). The highlight of stem cell research has been the design of feeder-free, xeno-free culture systems with chemically defined media formulations. In this study, the major difference between HN4 line and P96 line existed in the feeder cells used for derivation of hESCs lines in additional to the genetic factor. The maintenance and propagation of HN4 line and P96 line hESCs were both performed by feeder-free culture system based on mTeSR 1 medium using Corning Matrigel as the surface coating matrix (Ludwig et al., 2006). The above-mentioned feeder-free culture system is widely used for the maintenance of hESCs, but seldom used for derivation of hESCs lines because feeder-free derivation might induce karyotype abnormalities of hESCs (Draper et al., 2004; Ludwig et al., 2006). Therefore, Systematic analysis of the properties of hESCs derived on different feeders cells are necessary to decide which feeder cells are optimal.

Feeder cells play a dual role in maintaining hESCs morphology and pluripotent status in feeder-dependent culture conditions. Different cell types in feeder layers are able to secrete various growth factors to maintain the pluripotency and non-differentiation of growth of hESCs. They sustain hESCs attachment through expression of adhesion molecules and production of extracellular matrix (ECM). On the other hand, they also maintain the growth and survival of hESCs through secretion of growth factors. In this way, feeder cells may provide a favorable microenvironment for the growth and differentiation of hESCs. Since the culture conditions can influence gene expression that confers pluripotency in hESCs, it is necessary to investigate the impact of feeder cells on differentiation. Previously studies have shown that MEF feeder cells support cardiac differentiation better than HFFs feeder cells (Ojala et al., 2012; Pekkanen-Mattila et al., 2012). Here, we first reported whether the feeder cell type has an effect on dopaminergic differentiation of hESCs from different aspects, including neural induction process, differentiation potential, morphology and electrophysiological properties. In this study, MFCs feeder cells, a mixture of HFFs and MEFs, were used for derivation of hESCs to minimize the impact of culture condition variation on dopaminergic differentiation. MFCs feeder cells were used for successful establishment of hESCs lines in previous studies. The optimal mixture ratio of HFFs and MEFs was 1:1(Li et al., 2010; Yang et al., 2016).

In this study, both HN4 and P96 cell line could be differentiated into dopaminergic neurons. Moreover, the difference in feeder cells had no effect on neural induction process of dopaminergic differentiation and morphogenesis of dopaminergic neurons. We established a monolayer differentiation method using dual inhibition of SMAD signaling to promote efficient neuronal differentiation (Chambers et al., 2009; Cooper et al., 2010). The small molecules DM and SB were able to initiate neural induction of hESCs through the up-regulation of Wnt1-LMX1A levels, but accompanied by a reciprocal down-regulation of SHH-FOXA2 levels (Morizane et al., 2011; Cai et al., 2013). Exogenous SHH/FGF8/Vitamin C balanced in the aforementioned two pathways to induce the correct dopaminergic phenotype in ventral midbrain neural precursors (Cooper et al., 2010; Cai et al., 2013; He et al., 2015). Neuronal maturation was induced by IGF1, BDNF, GDNF, and cAMP (Chambers et al., 2009; Ning et al., 2009; Cooper et al., 2010). Our results revealed that different feeder cells didn’t promote or delay neural differentiation process, and the morphology changes of differentiated cells of HN4 line were similar to those of differentiated cells of P96 line. We further observed the morphology and ultrastructure of hESCs-derived neurons with TEM and SEM. A9 DA neurons were characterized by large angular or fusiform somas (Thompson et al., 2005; Bye et al., 2012). In addition, the ultra-structure of typical synaptic structure and numerous synaptic vesicles exhibited the maturity of hESCs-derived neurons in this study. The results suggested that hESCs cultured on different feeder cells could be differentiated into mature neurons with typical morphology and ultrastructure completely.

A notable difference between the two cell lines was the dopaminergic differentiation potential. Our study demonstrated that hESCs cultured on HFFs were more efficient source of neural progenitors than hESCs cultured on MEF feeder cell layers. Based on immunocytochemistry, the dopaminergic differentiation efficiency of P96 cell line was higher than that of HN4 cell line. Previous studies reported that various hESCs lines expressed similar markers in the undifferentiated state; however, these marker expressions might become variable once the cells began to differentiate. (Osafune et al., 2008). Each individual hESCs line has a unique gene expression profile (Abeyta et al., 2004; Skottman et al., 2005) and existed the differences in line-specific epigenetic profiles (Gan et al., 2007; Kim et al., 2007). The variability in differentiation capacity has been attributed to the unique gene expression of each cell line and line-specific epigenetic profiles. Different hESCs lines exhibit a marked propensity to differentiate into specific lineages, for example, HUES 8 is best for pancreatic differentiation and HUES 3 for cardiomyocyte generation (Osafune et al., 2008). In addition, differences in culture conditions also have a considerable influence on the gene expression profile and subsequent characteristics of hESCs. For example, serum- and feeder cell-free culture conditions, as well as the processes of enzymatic passaging and culturing of hESCs in physiological normoxia (2%), have been found to alter the gene expression profile and epigenome of hESCs (Skottman et al., 2006; Allegrucci et al., 2007; Forsyth et al., 2008). The microenvironment of stem cells provided by feeder cells may regulate stem cell self-renewal, proliferation and differentiation via external signals (Lu et al., 2006). Previous studies suggested that human feeder cells facilitated differentiation of hESCs into definitive endoderm formation (Zhou et al., 2008). hPSCs cultured on a Matrigel matrix in mTeSR1 medium were found to be more committed to neural lineage than hPSCs cultured on MEF or SNL feeder cell layers (Ojala et al., 2012). We assumed that our result is due to the fact human feeder cells have the ability to produce bFGF, while MEFs do not secrete bFGF. bFGF has been found to play a role in the derivation, proliferation and maintenance of the neural progenitor state (Eiselleova et al., 2008; Ma et al., 2012; Yang et al., 2016).

Another notable difference between the two cell lines is electrophysiological function of hESCs-derived DA neurons in this study. The electrophysiological excitability of P96-derived DA neurons was higher than that of HN4-derived DA neurons. The electrophysiological criteria of DA neurons in substantial nigra compacta *in vivo* and *in vitro* were established: (1) fired spontaneous APs in the range l–8 Hz, or quiescent; (2) long-duration APs (>2 ms); (3) a comparatively depolarized thresholds for activation (i.e., -30 to -45 mV), and a marked after hyperpolarization (Grace and Onn, 1989; Lacey et al., 1989). Our data demonstrated that DA neurons from HN4 and P96 cell line were both mature, functional DA neurons because they expressed voltage-gated sodium and potassium channels, ligand-gated GABA and Glutamate channels, and fired APs and Spontaneous PSCs, consistent with some other reports (Donato et al., 2007; Sagal et al., 2014; Stanslowsky et al., 2014). More importantly, they met electrophysiological criteria of dopaminergic neurons. However, the differences in electrophysiological properties were obvious between HN4- and P96-derived DA neurons. First of all, the activated potentials of voltage-gated sodium channels of DA neurons were different between the two cell lines. In this study, current-voltage plot showed that *I*_Na_ started to activate at around -60 mV or -50 mV and peaked at around -20 mV or -30 mV in P96- and HN4-derived DA neurons, respectively. Previous studies reported that activated potentials of voltage-gated sodium channels of from hESCs-derived DA neurons reported in were -60, -50, and -40 mV (Seutin and Engel, 2010; Ding et al., 2011; Stanslowsky et al., 2014). The differences between the two cell lines suggested the voltage-gated Na^+^ channels of P96-derived DA neurons were activated more easily than that of HN4-derived DA neurons. Secondly, the average Glutamate-induced currents amplitude of P96-derived DA neurons was higher than that of HN4-derived DA neurons. Our results demonstrated that both of hESCs-derived DA neurons expressed different types of receptor-operated channels in response to the excitatory and inhibitory neurotransmitters Glutamate and GABA, similar to some previous studies (Schulz et al., 2004; Hermann et al., 2006; Vazin et al., 2009). The difference in Glutamate-induced currents might be associated with the excitatory glutamate receptors density of P96- and HN4-derived DA neurons. Most importantly, the differences in electrophysiological parameters of AP of DA neurons between the two cell lines were quite obvious. Moreover, the specific repetitive firing pattern in response to currents injection was found in both of hESCs-derived DA neurons. In this study, duration and spike threshold potential of APs were 5.8 ± 0.24 ms and -39.58 ± 0.17 mV for P96-derived DA neurons, whereas it was 4.45 ± 0.08 ms and -36.15 ± 0.34 mV for HN4-derived DA neurons. RMP of HN4- and P96-derived DA neurons is -63.38 ± 0.01 and -58.7 ± 0.04 mV, respectively. The difference between RMP and spike threshold potential was smaller P96-derived DA neurons in comparison with HN4-derived DA neurons. The result suggested that DA neurons from hESCs cultured on HFFs were activated by external exciting signals.

In this study, both of hESCs-derived neurons fired in specific repetitive firing patterns in response to depolarizing currents: a progressive increase in AP duration, a decline in AP amplitude. This type of repetitive firing pattern were termed burst firing pattern, which met the “80/160 ms” criteria (Grace and Bunney, 1984a). The linear relationship between the number of spikes of AP traces and the intensities of the depolarizing currents was consistent with mesencephalic dopamine neurons in primary dissociated cell culture (Chiodo and Kapatos, 1992). Previous study thought that burst firing pattern of DA neurons was only observed *in vivo* (Kuznetsov et al., 2006). However, other studies have proven that primary DA neurons derived from embryonic mice, neonatal rats exhibited burst firing spontaneously or evoked by depolarizing injection current *in vitro* (Chiodo and Kapatos, 1992; Schulz et al., 2004; Blythe et al., 2009). A possible reason for this discrepancy is that intracellular recordings *in vivo* caused relatively greater damage to the somatic membrane than *in vitro* intracellular or patch-clamp recordings.

Similar to the results of our study, other hPSCs from different sources, such as human induced pluripotent stem cells (hIPSCs) and human amnion epithelial cells (hAECs), have been shown to be differentiated into fully functional DA neurons *in vitro* (Broughton et al., 2012; Li et al., 2015). The high survival and integration rates of transplanted cells are the key factors for the success of transplantation after intra-cerebral transplantation, depending on local microenvironment. So far, there have been many reports that neuroinflammatory processes were implicated with PD pathogenesis (Xu et al., 2012; Chen et al., 2015). Toll-like receptor 4 (TLR4), nerve growth factor IB (Nur77) and Nuclear receptor related 1 (Nurr1) played a critical role in the induction of neuroinflammatory responses in PD model (Gao et al., 2016; Wei et al., 2016; Zhou et al., 2016). neuroinflammatory responses in PD pathogenesis might affect the survival rate of transplanted cells *in vivo*. The use of neuroprotective agents, such as simvastatin, provided robust neuroprotection against dopaminergic neurodegeneration induced by neuroinflammation (Xu et al., 2013). Therefore, it is possible that DA neurons transplantation combined with neuroprotective treatment can improve the transplant outcome.

In summary, the present study suggests that both of hESCs lines cultured on HFFs and MEFs could be differentiated into functional, mature DA neuron. However, we first reported that HFFs feeder not only prompted the differentiation of hESCs cells into dopaminergic neurons, but also induced hESCs-derived DA neurons to express higher electrophysiological excitability. Future prospective studies are needed to investigate whether the differences in electrophysiological properties of different hESCs-derived DA neurons have an impact on transplant outcome *in vivo*.

## Author Contributions

ZZ and YM: conception and design, collection and assembly of data, data analysis and interpretation, manuscript writing, final approval of manuscript. QiaL: data analysis and interpretation, manuscript writing, final approval of manuscript. QiL, DK, KY, LH, TW, XC, YP, and WJ: collection and/or assembly of data, final approval of manuscript. ZC, YY, and XL: conception and design, financial support, final approval of manuscript.

## Conflict of Interest Statement

The authors declare that the research was conducted in the absence of any commercial or financial relationships that could be construed as a potential conflict of interest.
